# The Role of RNA Secondary Structure in Regulation of Gene Expression in Bacteria

**DOI:** 10.3390/ijms22157845

**Published:** 2021-07-22

**Authors:** Agnieszka Chełkowska-Pauszek, Jan Grzegorz Kosiński, Klementyna Marciniak, Marta Wysocka, Kamilla Bąkowska-Żywicka, Marek Żywicki

**Affiliations:** 1 Department of Computational Biology, Institute of Molecular Biology and Biotechnology, Adam Mickiewicz University, Uniwersytetu Poznańskiego 6, 61-614 Poznań, Poland; agnieszka.chelkowska@amu.edu.pl (A.C.-P.); jan.kosinski@amu.edu.pl (J.G.K.); marta.wysocka@amu.edu.pl (M.W.); 2 Institute of Bioorganic Chemistry, Polish Academy of Sciences, Noskowskiego 12/14, 61-704 Poznań, Poland; kmarciniak@ibch.poznan.pl

**Keywords:** RNA structure, regulation of gene expression, ncRNAs, riboswitches, bacterial sRNAs

## Abstract

Due to the high exposition to changing environmental conditions, bacteria have developed many mechanisms enabling immediate adjustments of gene expression. In many cases, the required speed and plasticity of the response are provided by RNA-dependent regulatory mechanisms. This is possible due to the very high dynamics and flexibility of an RNA structure, which provide the necessary sensitivity and specificity for efficient sensing and transduction of environmental signals. In this review, we will discuss the current knowledge about known bacterial regulatory mechanisms which rely on RNA structure. To better understand the structure-driven modulation of gene expression, we describe the basic theory on RNA structure folding and dynamics. Next, we present examples of multiple mechanisms employed by RNA regulators in the control of bacterial transcription and translation.

## 1. Introduction

In the course of evolution, bacteria have developed many mechanisms enabling a rapid adaptation to environmental conditions via instant regulation of gene expression. Among them, the fastest and most efficient is the employment of regulators based on modulation of the RNA structure. It is possible due to the intrinsic RNA’s ability to fold into sophisticated structures through the base pair interactions among distantly located regions as well as by pairing local nucleotides into hairpins. These structures are highly dynamic and can comprise motifs as simple as base-paired double-stranded regions that form in trans or cis [1,2], and as complex as intricate tertiary structures such as pseudoknots, ribose zippers, and kink turns [3]. These motifs can also assemble into larger independent folding domains and multi-molecular complexes.

Dynamically changing RNA structure is involved in the control of the majority of gene expression steps, from transcription, through translation, up to RNA decay [4,5]. In prokaryotes, individual helical regions, typically in the 5′ untranslated regions (UTR) or early in the coding sequences, are critical for control of transcription and translation by modulation of signals for RNA polymerase and the ribosome [6,7]. These regions are often accompanied by much more complex structures, e.g., aptamers of riboswitches, that provide the environment sensing and can switch the base-pairing between mutually exclusive folds to sequester or expose regulatory elements [8,9].

The topic of RNA structures in different cellular processes has been covered in several excellent reviews [10,11,12,13,14]. Therefore, in this review, we will highlight the influence of the conformational alterations in the secondary and tertiary ribonucleic acid structures on enhancing or attenuating downstream events in the course of bacterial transcription and translation.

## 2. Dynamics of RNA Structure as a Source of Regulatory Potential

The versatility of RNA in forming both simple and complex structures allows it to engage in diverse interactions with other molecules, ranging from base-pairing with small RNAs to forming binding pockets for cations (e.g., Mg^2+^, K^+^) and complex metabolites (e.g., vitamins, coenzymes, nucleotide derivatives, amino acids), as well as binding structural, processing, and signaling proteins [15,16,17]. All the RNA molecules can undergo structural transitions. Under particular conditions, they are stabilized or destabilized, and in most cases, the different structures are coexisting in the cell at a given equilibrium. This tendency of RNA molecules for highly dynamic structural changes on a microsecond time scale [18] provides the enormous regulatory potential to control the adaptation of bacteria to changing environments.

### 2.1. RNA Free Energy Landscape

**Figure 1:**
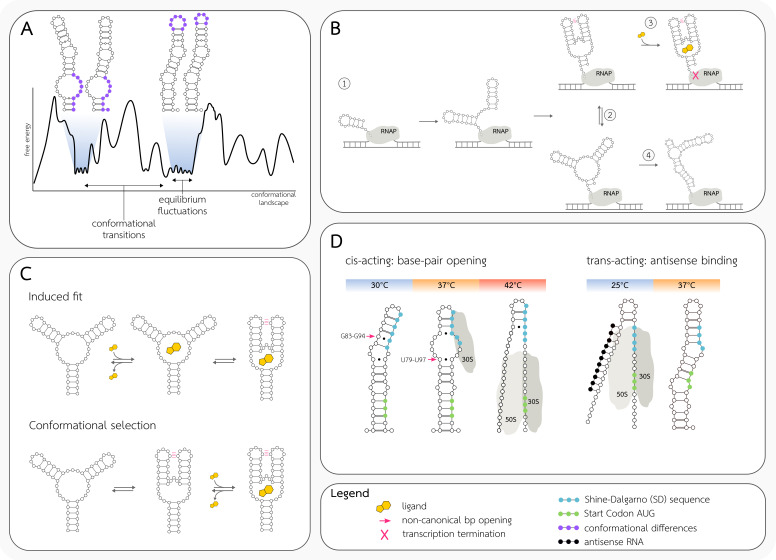
RNA secondary structure dynamics. (**A**) The conformational landscape of RNA. Detailed structural conformation examples are shown for two energy minima within the landscape. The differences between structures within one energy minima are shown in purple. (**B**) Schematic representation of riboswitch co-transcriptional folding. (1) Preliminary folding of the 5′ end of RNA during the transcription. (2) Synthesized riboswitch aptamer can fold into two conformations. One of them, stabilized by ligand binding (3), leads to transcription termination. The second does not inhibit the process and undergoes further conformational changes along with the RNA synthesis (4). (**C**) Two mechanisms of ligand binding and conformation stabilization of the riboswitch. The induced fit assumes that the aptamer conformational change is due to the ligand binding. In conformational selection, ligand binding stabilizes dynamically changing aptamer conformations. (**D**) RNA thermo-sensors representation. In the cis-acting thermo-sensor ROSE (Repression Of heat Shock gene Expression) (left), the structural change is introduced by the non-canonical (shown with dot as binding) base-pair openings induced by the increase of the temperature. The disrupted base-pairings (shown with an arrow) allow the structure to be unraveled and the binding of the ribosome. In the trans-acting thermo-sensors, the antisense RNA (synthesized in the cell at low temperatures) binds to the mRNA, allowing the ribosome binding and translation initiation. At higher temperatures and absence on the antisense RNA, the Shine-Dalgarno sequence and AUG codon are inaccessible to the ribosome.

The spatial arrangement of RNA molecules is defined by self-interactions as well as interactions with other molecules or the environment. RNA secondary structure tends to minimize the Gibbs free energy of the system, but the RNA sequence does not fold into a single, energetically favorable secondary structure [19]. The structure of RNA can be described as a distribution of numerous conformations which match the sequence pattern. Each of the conformations, called intermediate structures, has its free energy, which together creates an energetic landscape of RNA structure ensemble (Figure 1A) [20]. Since the low free energy, and hence thermodynamic stability, is preferred, the conformations’ populations are not equal. Some conformations are more abundant in the ensemble, and together with the energetically close structures create the energy minima—suboptimal structures within the landscape. Since some conformations matching the sequence model cannot obtain adequate energy stability, the RNA landscape is dominated by only a few conformations, and the rest of them are rather unstable intermediate forms [21].

### 2.2. Free Energy Barriers

The transition between conformations depends on the height of the energetic barrier. This barrier describes the complexity of the structural changes and the free energy, which have to be provided into the system to convert between two considered structures [22,23]. Conformations within the same local energy minima tend to have a lower barrier than those between different minima [21,24]. This leads to the formation of the kinetic traps, where the barrier is too high to promote the structural rearrangements and the structure is captured in the non-optimal local minima conformation. An example of such behavior is the splicing of the T4 phage-derived *td* intron in *Escherichia* *coli* [25]. The fragment of the 3′ sequence of intron forms a stable hairpin with the complementary sequence of the upstream exon, preventing the splicing. This conformational trap can be resolved by providing additional energy factors to the system, in this case by binding the ribosome to the upstream gene, or by opening the misfolded structure by RNA chaperones (StpA) [26]. Such induction of structural rearrangements by external factors providing energy for structural transitions is a primary mechanism employed by RNA regulators.

### 2.3. Types of RNA Conformation Dynamics

Dethoff et al. [21] define two types of RNA dynamics: equilibrium fluctuations and conformational transitions (Figure 1A). The first type, equilibrium fluctuations, relates to the continuous and spontaneous conformation changes through the free energy landscape. The rate of those fluctuations depends on the level of the free energy barrier separating the conformations. These motions mostly occur in the free energy minima within the landscape. Spontaneous transitions between different local minima can occur only in the case of RNA molecules in which two alternative folds are separated by a low-energy barrier. Such bi-stable molecules usually exist in close to 50:50 structural equilibrium, until a stabilizing factor binds to one of the conformations, shifting the equilibrium.

The conformational transition allows the changes in the conformation of RNA between the energy minima of the free energy landscape. The conformational transition occurs when the free energy landscape is disrupted by cellular changes, e.g., the change in metabolite concentration [14,27] or the temperature [28]. Such cellular cues provide the free energy shift necessary to unfold the RNA region and overcome the energy barrier. As a result, the conformational ensemble is redistributed, and the new folding pathway becomes available, which includes the interacting factor leading to stabilization of the new conformation. These conformational changes are temporary, and after the factor release, the ensemble distribution returns to equilibrium [21].

### 2.4. Co-Transcriptional Folding

In the early 1980s, it was found that the nascent RNA folds into secondary structures as they are transcribed in the cell [29,30], rather than after the completion of the transcription process. Such a dynamic process directly affects the crossing of energy barriers and thus facilitates the conformational transformation of the RNA molecule [31]. Therefore, the co-transcriptional folding occurs sequentially from the 5′ end to the 3′ end of RNA, and the newly formed RNA starts to fold before the 3′ part of the chain is synthesized (Figure 1B). Schroeder et al. [32] listed three implications of transcription on the RNA structure folding. First, the order of RNA folding is conditioned by the direction of the transcription process, which promotes the dynamic local interactions of newly synthesized RNA. Second, the RNA polymerase velocity differences in the rate of transcription by different polymerases will directly affect RNA folding. The velocity of bacteria polymerase is around 20–80 nucleotides per second, but the RNA folding occurs in a wider range of time, from 10-100 msec to even minutes or hours for kinetically trapped conformations [33]. The third effect of transcription on the folding of the RNA structure is transcription pausing. In bacteria, the interaction of synthesized RNA and polymerase and/or protein factors can induce transcription pausing [34,35,36]. An example of this behavior is a flavin mononucleotide (FMN)-dependent riboswitch from the *ribDEAHT* operon of *Bacillus subtilis*. The metabolite binding, stabilizing one of two possible co-transcriptional conformations, occurs within the polymerase pausing, resulting in the formation of the terminator stem [37].

### 2.5. Riboswitch Structure Dynamics

Riboswitches are cis-acting RNA structures that regulate bacterial transcription as well as translation, usually located in 5′ leader regions of the mRNA molecules (reviewed in [16,38,39,40,41]) and evolutionary conserved through the kingdoms of Bacteria, Archaebacteria, and rarely Eukaryota [16,42]. Their role is to act as RNA-based sensors of small molecules or ions. Ligands are known to bind an aptamer region of the riboswitch, inducing its structural alterations which are propagated to the expression platform, usually located downstream of the aptamer [38,41]. Changes in the structure of the expression platform result in the initiation of one out of several different mechanisms responsible for the regulation of gene expression. The strategy for ligand binding and modification of the expression platform will depend on the concentration of the target metabolite in the cell. In their recent work [27], Scull et al. described two models of the ligand binding: a conformation selection and an induced fit (Figure 1C). The conformation selection mechanism occurs when the ligand binding is preceded by a change in the aptamer conformation between the two states or into an intermediate form. In this scenario, the aptamer is dynamically switching the conformations and the ligand-binding enables the stabilization of one state and changes in the expression platform. Alternatively, the induced fit mechanism is present when the ligand-binding occurs before the conformation change in the aptamer.

The fluoride sensing riboswitch represents the conformation selection mechanism of ligand binding. It was observed that this riboswitch folds into a single structure, regardless of the concentration of the F-. However, in the absence of F-, the short-living intermediate conformation with an accessible ion binding site can be observed (so-called “excited state”). The binding of the F-, in a narrow concentration range, stabilizes this structure and triggers the conformational changes in the expression platform [27]. An example of an induced fit mechanism is a guanine sensing riboswitch in which the conformational changes in three-way junctions appear with the ligand binding in the center of this junction [43]. Some riboswitches exploit both mechanisms, e.g., the transcriptional preQ1 riboswitch from *B. subtilis* favors the conformational selection mechanism, where the ligand binds to pre-selected conformation. However, in the low concentration of the ligand and Mg^2+^, this riboswitch can apply the induced fit mechanism, where the conformational change begins with the binding [27].

### 2.6. RNA Thermometer Dynamics

RNA thermo-sensing structures, called RNA thermometers, undergo temperature-induced conformational changes or processing transformations to control the gene expression in bacteria. We can distinguish two mechanisms of thermoregulation: cis-acting translation initiation regulation and trans-acting antisense binding (Figure 1D). Cis-acting regulation is based on melting of the base-pairing regions near the Shine-Dalgarno and the start codon region. The trans-acting one requires the antisense sequence that disrupts the double-stranded regions related to the ribosome binding.

An example of the mechanism of a cis-acting switch is ROSE (Repression Of heat Shock gene Expression), present in the 5′ UTR in Gram-negative bacteria. ROSE controls the translation of the expression of small heat shock genes, such as the inclusion body binding protein A gene *ipb*A in *E. coli* [44]. RNA thermometers do not change conformation as other switches do. Their mechanism is based on the loops and weak bonds in the ribosome binding region, which are sensitive to temperature [45]. In their work, Chowdhury et al. [46] showed the mechanism of MiniROSE conformational change upon the temperature. At 30 °C, the Shine-Dalgarno (SD) sequence and the start codon of the mRNA are base-paired with the complementary sequence in the 5′UTR of the transcript, hence preventing the ribosome binding and translation initiation. As the temperature rises to 37 °C, the SD-adjacent region starts to destabilize, making it partially accessible to the 30S subunit of the ribosome. A further increase in the temperature causes the disruption of bonds in the SD and start codon regions, strengthening the mRNA–ribosome interaction. This mechanism is based on the weak hydrogen bonds in the region preceding the SD sequence—a triple internal loop (C80, U81, U96) forming a temporary hydrogen bonding network, with two noncanonical closing base pairs, U79-U97 and G83-G94 (Figure 1D). At 37 °C, the U79-U97 pair is affected, and further, in heat shock at 42 °C, the remaining weak base-pair (G83-G94) is destabilized as well [46].

The documented example of thermoregulation by trans-acting antisense binding is the RNA polymerase sigma factor S (RpoS) regulation in *E. coli*. A higher expression of RpoS is observed at 25 °C, causing the activation of RpoS-dependent promoters at lower temperatures. Expression of RpoS is regulated by the 85-nucleotide small noncoding RNA DsrA, the concentration of which is also higher at 25 °C. DsrA RNA takes two forms: the primary transcript F form, which is then further processed into a 60–61-nucleotide T form, lacking the 5′-region interacting with the *rpoS* mRNA. Since the primary transcript (F form) is more stable at 25 °C, the interaction with the leader sequence of the *rpoS* transcript is enabled. By binding the antisense RNA (5′ region of DsrA), the disruption of the base pairing in the flanking SD region occurs [47]. The pairing between the DsrA and *rpoS* mRNA is accelerated by the Hfq protein binding, which stabilizes the tertiary interactions of those RNAs [48]. The *rpoS* mRNA changes the conformation and activates the translation process, resulting in the increased expression of RpoS at low temperature.

## 3. Regulation of Transcription by Changes in the RNA Secondary Structure

Transcription termination plays an important role in the regulation of gene expression, allowing recycling of the RNA polymerase (RNAP), preventing transcription of the neighboring genes, and collisions with other transcriptional complexes. In bacterial genomes, two different modes of the termination process are distinguished. The first is based solely on the interaction between RNAP and DNA-template sequence (so-called intrinsic or Rho-independent termination), while the second involves cooperation with specific factors, such as Rho or Mfd proteins (called factor-dependent termination). In this review, we will only briefly describe factor-dependent transcription termination mechanisms as they are not directly regulated by RNA secondary structures.

### 3.1. Intrinsic Transcription Termination

**Figure 2:**
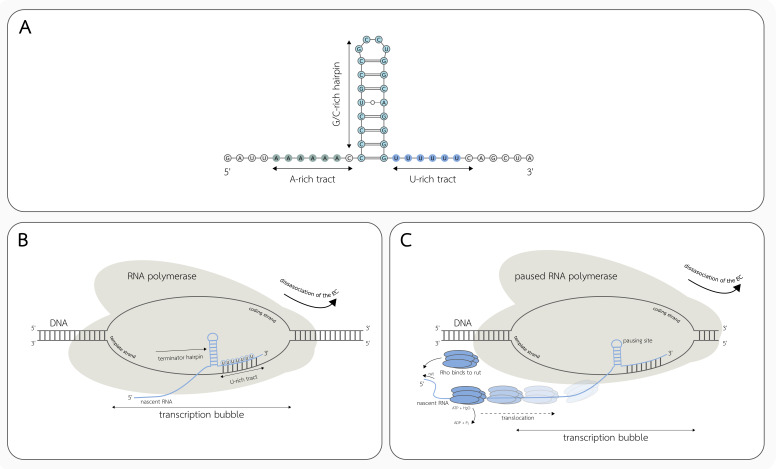
Regulation of transcription by changes in the RNA secondary structure. (**A**) Bidirectional intrinsic terminator composed of G-C-rich hairpin structure and immediately followed by a 7–8 nucleotide long U-rich tract. Here, the terminator hairpin is also preceded by the A-tract—a feature characteristic for bidirectional intrinsic terminators. (**B**) Schematic representation of the rho-independent termination in bacteria. RNAP pausing at U-tract, followed by the formation of the terminator hairpin in the nascent RNA molecule causes destabilization and disassociation of the elongation complex (EC). (**C**) Schematic representation of the rho-dependent termination mechanism. After Rho binds the U-tract, it is translocated towards the paused EC. Rho translocation is initiated by ATP hydrolysis. Interaction between the EC and Rho results in destabilization and disassociation of the RNAP and release of the nascent RNA molecule.

Intrinsic termination is associated with the formation of the G-C-rich hairpin structure (terminator hairpin) on the nascent RNA molecule, within 7–9 nucleotides (nt) upstream from the RNA 3′ end [49,50]. Importantly, the stem-loop at its 3′ terminus is known to be immediately followed by a 7–8 nt long region highly enriched in the uridine nucleotides (U-tract) [49,50,51]. Terminator hairpin can also be preceded by the A-tract (similarly to the U-tract), typical for bidirectional intrinsic terminators (Figure 2A) [52]. The U-rich segment is believed to cause RNAP pausing at its end, therefore allowing the formation of the stem-loop structure. It was also proposed that it might not be the U-tract itself that causes RNAP pausing, but the elemental pause sequence present in the terminator (reviewed in [49]). RNAP pausing and subsequent formation of the terminator hairpin favors the destabilization and ultimately the disassociation of the transcription elongation complex and the release of the nascent RNA (Figure 2B) [50].

It is still under debate as to what extent the features, described above as essential for efficient intrinsic termination, are conserved across all bacteria. Most of the research is based on *E.* *coli*, *B.* *subtilis,* and only a few other species, therefore leaving a huge variety of different microbial lineages relatively unstudied in terms of transcription termination regulation. As an example, Ahmad et al. [53] showed that the U-tract following the termination hairpin may not be required for an efficient termination in mycobacteria and a significant number of predicted terminators lack the canonical sequence of uridine nucleotides in the nascent transcripts. Additionally, researchers suspect that the U-tract is probably not the only determinant of RNAP pausing, and nucleotide sequences in the vicinity, e.g., in the RNAP exit channel, active site, or DNA channel, may also play a role in the efficiency of the process [50].

Roberts [49] in his review describes possible mechanisms of the RNAP destabilization. The forward translocation model assumes that after the termination, RNAP continues its operation across the DNA strand, but without extending the nascent RNA molecule, which results in the shortening of the RNA:DNA hybrid and favors disassociation of the elongation complex. The subsequent mechanism, called the slippage, is based on the extraction of the nascent RNA from the complex caused by the hairpin formation. Additionally, the allosteric model has been proposed, established on the idea that conformational change in the RNAP structure, caused by the hairpin formation, results in the opening of the elongation complex, ultimately leading to the collapse of the transcriptional bubble.

Although, in general, the intrinsic termination does not require any additional factors to cause the transcription termination, some of the factors have the potential to increase or decrease the efficiency of transcription termination. The most widely studied elongation factors, NusA and NusG, are relatively conserved across bacteria and archaea. They both stimulate the termination process by extending or introducing new RNAP pauses respectively, and increasing RNAP processivity [53,54,55,56]. As shown by Mondal et al., a significant number of the weak non-canonical intrinsic terminators might be strongly dependent on the NusA [54].

### 3.2. Factor-Dependent Transcription Termination

Rho- and Mfd-dependent termination processes belong to the distinct class of transcription events. In contrast to the intrinsic termination, those mechanisms are dependent on auxiliary factors such as Rho or Mfd translocases. Mfd-dependent termination is devoted to the recycling of the stalled elongation complexes that otherwise prevent DNA replication. Mfd binds to the RNAP and the upstream DNA sequence and initiates the translocation of the stalled complex, leading to the release of the nascent RNA molecule and the disassociation of the RNAP from the DNA template strand [50]. Rho is a homohexameric helicase, strongly conserved across the bacteria kingdom, not present within only a few lineages. These include *Mollicutes*, *Cyanobacteria*, and some species within *Clostridia* and *Bacilli* classes [57]. Rho is estimated to be involved in a significant number of termination events in *E. coli*. Washburn and Gottesman in their review [58] state that approximately 20% of the transcripts are terminated using this mechanism, and Mitra et al. [57] declare that it makes up about 50% of all termination events. Rho-dependent termination (Figure 2C) requires the presence of the rho-utilization (rut) sequence, which is recognized and bound by the Rho [49,59]. The rut site lacks any particular RNA secondary structure and comprises a sequence that is rich in cytosine and poor in guanine nucleotides [49,50,57,58,59]. Rho translocase activity is initiated by the ATP hydrolysis right after its binding to the rut site, inducing the Rho movement towards the 3′ end of the RNA molecule [58,59]. Termination is caused by “catching” the elongation complex paused at so-called release sites [50,58]. Transcription termination mediated by the Rho protein is known to be occasionally induced by changes in the RNA structure of various regulatory elements, for example, riboswitches (example can be found in Section 3.3) [60].

### 3.3. Conditional Terminators

Some of the transcription termination mechanisms existing in bacteria can terminate the process conditionally, in response to environmental factors. Here, we list the most prominent examples of such mechanisms, also providing several important reference articles, allowing the reader to further explore the topic in detail.

Riboswitches are RNA structures allowing bacteria to conditionally terminate transcription of the controlled gene(s) in response to binding a small molecule or ligand (described in Section 2.5). Riboswitch-dependent transcription regulation usually involves the formation or disruption of the intrinsic terminator (Figure 2), but in some cases can even affect the activity of the Rho helicase [60]. Formation of the intrinsic terminator hairpin is induced by conformational changes in the aptamer domain, which involves elements of anti- or anti-anti-terminator structures [13].

An example of activating Rho-dependent termination by Mg^2+^ sensing riboswitch mgtA in *Salmonella enterica* was described by Hollands et al., where in low Mg^2+^ concentration, the RNA region folds into the structure which covers the RUT site, promoting the expression of the *mgtA* gene. However, high Mg^2+^ induces refolding, releasing the RUT site, especially the R1 region, and making it accessible to Rho protein [60,61,62]. Then, Rho, using ATP, translocates along with the transcript towards the RNAP and causes transcription termination. There are indications that this process can also be modulated by the translation speed of the proline-rich MgtL leader peptide, which is Mg^2+^-sensitive. In this model, the presence of ribosomes and their pausing caused by low Mg^2+^ are the major factors causing the structural release of the RUT site [61]. The most likely is, however, that both mechanisms cooperate in *mgtA* regulation.

**Figure 3:**
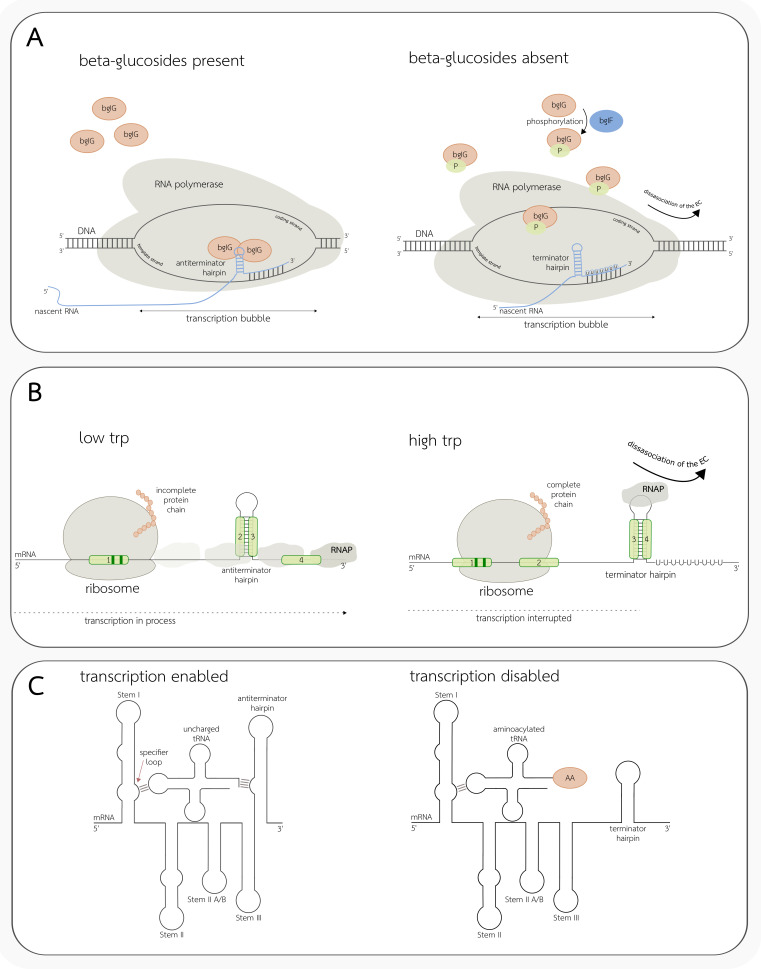
Selected mechanisms of conditional transcription termination in bacteria. (**A**) Regulation of the *bgl* operon in *E. coli* by transcription attenuation. In the absence of the β-glucosides (right side), protein bglF phosphorylates and sequesters protein bglG. Phosphorylated proteins cannot bind the anti-terminator site and block the formation of the intrinsic terminator, which can be observed while β-glucosides are present and bglG proteins are not phosphorylated (left side). (**B**) Regulation of the *Trp* operon in *E. coli* by ribosome stalling. The leader region of the operon contains four segments (1, 2, 3, and 4). The 1st segment overlaps with the short ORF containing Trp codons (marked in dark green color). Low Trp levels (left side) cause ribosome delay during the translation of the Trp codons. Stalled ribosome covering the 1st segment induces the formation of the anti-terminator hairpin containing segments 2 and 3. While the level of Trp is sufficient (right side), the ribosome can continue translation and cover both 1st and 2nd segments, disrupting the anti-terminator, which leads to the formation of the competitive hairpin containing segments 3 and 4, which acts as a transcription terminator. (**C**) Schematic representation of the T-box structure. On the left side of the picture, uncharged tRNA binds to the specifier loop and interacts with the bulge of the anti-terminator, therefore stabilizing the structure so that the transcription process can proceed. On the right, charged tRNA attaches to the T-box, but interaction with the anti-terminator cannot be established because of the presence of the aminoacyl group (AA). This results in the formation of a competitive structure that causes transcription termination.

A similar mode of action is shared between riboswitches and so-called attenuators, the difference being in the cause of conformational change leading to the transcription termination or anti-termination. In the case of the attenuators, structural modifications might be facilitated either by the stalled ribosomes, RNA-binding proteins, or uncharged tRNA molecules [40,62]. As an example, RNA-binding proteins are crucial for the regulation of the bgl operon in *E. coli* (Figure 3A). Its expression is highly dependent on β-glucosides, and in their absence, protein bgIF phosphorylates and sequesters protein bgIG (in the presence of the substrate, a reverse process of dephosphorylation can be observed). Both proteins are products of genes controlled by the *bgl* operon. Phosphorylated bgIG is unable to bind the ribonucleic anti-terminator sites in the nascent RNA, where it is known to block the formation of intrinsic terminators otherwise present in the operon through the stabilization of the competitive secondary structure [62].

The tryptophan (*Trp*) operon in *E. coli* can be an example of transcription regulation by ribosome stalling (Figure 3B). Its leader region consists of four different segments. Every two of them located next to each other can form a hairpin structure together (1st with 2nd, 2nd with 3rd, and 3rd with 4th). The 1st segment (counting from the 5′ end of the leader region) partially overlaps with the short open reading frame (ORF) containing the Trp codons. The hairpin structure assembled from the 1st and the 2nd sections is known to act as a transcription pausing site. RNAP can resume the transcription process only after the ribosome disrupts the hairpin structure. A low concentration of the Trp in the cell results in translation delay at the Trp codons within the leader ORF, while transcription can proceed further to the end of the operon. Stalled ribosome prevents the formation of the first hairpin, which induces the assembly of the second (consisting of segments 2 and 3), acting as an anti-terminator. In contrast, while high levels of Trp are accessible, the ribosome can advance the translation and covers both the 1st and the 2nd segments, causing the formation of the intrinsic termination hairpin from sections 3 and 4, therefore preventing further transcription of the operon genes [62].

Uncharged tRNAs are the substrates known to regulate processes of conditional transcription termination of genes related to the metabolism of the amino acids [63]. mRNA structures binding tRNA molecules are called T-box riboswitches (Figure 3C) and are most abundant in Gram-positive bacteria. T-boxes are known to respond to amino acid starvation by sensing aminoacylation levels of tRNAs, therefore analyzing their readiness for translation [63,64]. T-boxes contain 2 or 3 different stem structures responsible for their function, followed by the competing terminator or anti-terminator helices [65]. Stem I, near its 5′ end, is responsible for binding specific tRNA ligand, which can be achieved by the presence of a so-called specifier region complementary to the tRNA anticodon [63]. Stem II (not present in all T-box structures) is capable of creating additional mRNA–tRNA contacts, which are believed to strengthen interactions with Stem I [63]. Finally, Stem III is believed to be involved in the stabilization of the anti-terminator structure, which is required for proper aminoacylation sensing [63,65]. The stabilization itself is enforced by the binding of the uncharged tRNA acceptor arm to the bulge of the anti-terminator, which prevents further structural folding into the mutually exclusive intrinsic terminator hairpin at the 3′ end of the T-box. The described interaction does not occur with the charged tRNA acceptor arm, thus the tRNA binding results in the formation of the alternative secondary structure, causing transcription termination [65].

## 4. Regulation of Translation by Changes in the RNA Secondary Structure

The strict control of protein synthesis is required under both, constant and variable environmental conditions to maintain the right level of proteins, which are essential for cell viability. The translation is the most energy-demanding process in cells so there is no place for dispensable reactions. Translation can be regulated at every step, including initiation, elongation, termination, and ribosome recycling, but the most studied is control at the initiation step [66]. The crucial step in the formation of an active translational complex is the isomerization step, leading to a transformation of the unstable pre-initiation complex to a stable initiation complex composed of 30S ribosomal subunit, mRNA, and tRNA-fMet. The efficiency of this irreversible process strictly depends on the ratio between the kinetics of isomerization and mRNA structure unfolding (for details see [67,68]). Thus, the most commonly exploited and most effective mechanisms of translation regulation rely on the modulation of structural accessibility of a SD and RBS [69]. The diversity of mechanisms used by RNA regulators at this very first step of translation is one of the reasons for the complexity of this process [70]. RNA regulators of translation can be roughly divided into two groups, cis- and trans-acting.

### 4.1. Cis-Acting Translation Regulators

Thermosensors and riboswitches are the most ubiquitous representatives of RNA structures regulating translation in cis. Three classes of bacterial genes are under temperature-dependent control—virulence, cold shock, and heat shock genes. In translation, thermosensors act by sequestering RBS in a secondary structure that responds to temperature fluctuation (Figure 1D). They can employ two different mechanisms: the zipper mode, where in response to temperature, melting of RBS-masking stem occurs and the SD sequence becomes accessible to the ribosome subunit [20,45,54,70]; or the switching mode, where in response to temperature change, conformational switching occurs releasing the SD sequence [45] (for details see Section 2.6 of this review). This first process can be observed in *Listeria monocytogenes* in response to its invasion into a host. At host body temperature thermosensor activates the translation of the *prfA* mRNA by releasing its RBS from a stem structure. This results in the expression of PrfA protein, which is the main regulator of the virulence in this bacteria [21,71]. Other examples of genes regulated by the zipper thermosensors are IbpA—an inclusion body-binding protein A in *E. coli*, hspA—*Bradyrhizobium japonicum* heat shock protein A, and small heat shock gene aggregation suppressing A (AgsA) in *Salmonella enterica*. On the other hand, a thermosensor with a switching mode was found in enterobacteria phage λ, where it changes the structure from A to B. When the temperature increases, structure A stabilizes and prevents the synthesis of cIII protein, promoting bacteriophage into the lytic cycle, and structure B enables translation of this protein. Thermosensors can also be triggered by temperature decrease, e.g., in control of the cold-stress response in *E. coli*. In this case, the cold-induced structural rearrangement causes the release of RBS from the stem, activating the synthesis of cold shock protein A (CspA) [45].

Even more complex translational regulators are riboswitches. Similar to RNA thermosensors, they control the initiation step by modulation of the RBS structural availability, however, the process is induced by binding the small ligands. [67,72]. As a result, translation can be either, inhibited or induced, by forming alternative mRNA structures that occlude or expose the SD sequence, respectively [4,21,28,40]. Translation modulation by riboswitches is more common in Gram-negative bacteria, in contrast to Gram-positive bacteria, where the predominant form of riboswitch regulation is transcription attenuation (for details see the previous section) [40]. Breaker distinguished three groups of translational riboswitches, dividing them according to the RBS localization. As a first type he described riboswitches in which RBS is an intrinsic part of an aptamer, thus the ligand and the ribosome compete for its binding. An example of this type of riboswitch is cobalamin binding riboswitch, where the high level of the ligand inhibits the translation. The second type contains separate ligand and RBS binding domains—aptamer and expression platform. A representative of such riboswitches is one of the most widely spread, thiamin pyrophosphate (TPP) binding riboswitches. These regulators change the conformation of the expression platform apart from the aptamer upon the binding of a TPP. The last type of riboswitch mechanism also found within the TPP riboswitch family, is a strict two-step regulatory system, where the RBS is localized in the stem which is also the rho-independent transcription terminator. In this type of riboswitch, the efficient binding of the ligand at a high concentration prevents transcription by the formation of a terminator structure. However, if the concentration of TPP is moderate and transcription can occur to full-length mRNA transcripts, the post-transcriptional regulation by occluding RBS into a double-stranded structure occurs [38].

Another interesting mechanism that involves riboswitches is the indirect regulation of the translation degradation of the mRNA. This phenomenon was discovered in Gram-positive bacteria, like *B.* *subtilis*, wherein the presence of a glucosamine-6-phosphate (GlcN-6-P), the glmS riboswitch activates the self-cleaving ribozyme in the 5′ UTR of transcript coding glutamine-fructose-6-phosphate amidotransferase (GlmS). Next, the coding region of the mRNA is rapidly degraded by RNase J1 [38,73]. Another example is lysine riboswitch in *E. coli,* which can preclude the translation by sequestering the RBS sequence into the secondary structure and the same riboswitch can target mRNA for degradation by RNase E [38,74].

RNA secondary structure can modulate translation also via induction of the ribosome pausing and collision. Increased pausing leads to lower efficiency of protein synthesis since the time needed for a single translation is longer and fewer proteins will be synthesized from single mRNA within its cellular lifetime. There are few reasons for the ribosome pausing, including strong mRNA secondary structures during translation initiation and ribosome translocation. Since ribosomes possess the helicase activity and can unwind the secondary mRNA structures, the pausing during translocation is rather temporary. Such pauses can lead to changes in translation efficiency only when many ribosomes are translating the same mRNA, creating polysomes. The situation, when pausing takes place on a polysome leads to the queuing of the ribosomes. In extreme cases, collisions of the ribosomes can occur, which are recognized in bacteria by the RqcH, a protein that is a part of the ribosome quality control system and marks the nascent protein chain to degradation by the ClpXP protease [66].

Also, the higher-order structures can be involved in translation regulation in bacteria. G-quadruplexes (G4s) are G-rich structural motifs, originally discovered in DNA, that can fold into a minimum of two stacked G-quartets (four guanines connected by H-bonding) and are stabilized by the potassium ions (K^+^). The same structure can be also formed by RNA (rG4s). A recent study showed that rG4s can be found in many bacteria species. Shao and colleagues revealed some more insights into the rG4s. They demonstrated that the distribution of these structures is species-specific and that rRNAs are less abundant in the form of quadruplexes. Moreover, they associated rG4s with important biological processes in *E. coli* and *Pseudomonas aeruginosa*, including regulation of metabolic processes and gene expression [75].

### 4.2. Trans-Acting Translation Regulators

**Figure 4:**
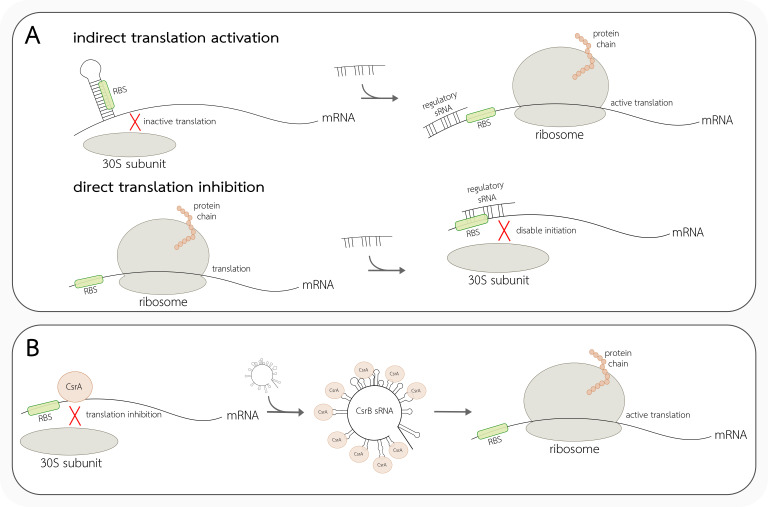
Examples of trans-acting regulation of translation. (**A**) Regulatory sRNAs regulate gene expression indirectly, by induction of RBS release, or directly, via masking the RBS region. (**B**) Regulation in Csr system. CsrA protein inhibits translation by binding nearby the RBS. CsrB sRNA has multiple binding domains for CsrA, thus it sequesters the protein and enables the ribosome subunit to initiate translation.

The RNA-dependent regulation of translation can also occur in a trans. It is fulfilled by binding of products of other genes expression, including proteins and non-coding RNAs [66]. In both cases, the regulatory effect is caused by direct or indirect masking of the RBS region by interacting factors. Translation regulation dependent on proteins consists of binding molecules within or nearby the RBS to modulate the interaction of 30S ribosomal subunit with the SD sequence or to induce conformational changes of mRNA which affect the RBS accessibility. In trans-acting ncRNA complementarity with its target mRNA is usually not perfect, thus the formed heteroduplex is similar in terms of structural plasticity and dynamics to regular RNA structures. Non-coding RNAs can interfere with the translation by binding target mRNA near the ribosome binding site, blocking its ability to form an initiation complex (Figure 4A). Trans-encoded sRNAs can also prevent the formation of RBS occluding hairpin and thus positively regulate translation (Figure 4A). Bacterial small RNAs are the most common group of such regulatory ncRNAs, mainly in response to environmental changes [40,70]. One of the first described ncRNA regulators was MicF RNA, sRNA found in *E. coli*, that inhibits translation of OmpF—the major outer membrane porin by masking the RBS site [40]. However, ncRNA-driven regulation can also be mediated by the propagation of dynamic structural changes within the target mRNA. An example of such ncRNAs is SR1, an sRNA that binds 100 bp downstream from the start site of *ahrC* mRNA in *B. subtilis*, which is the transcriptional activator of the two arginine catabolic operons. The binding induces the structural changes downstream of the SD sequence of *ahrC* mRNA and thus blocks ribosome binding to RBS and regulates arginine catabolism [68,76].

As mentioned earlier, ncRNA binding can also activate the translation. One of such regulatory processes, involving GlmZ and GlmY ncRNAs occurs co-transcriptionally taking advantage of the formation of intermediate structures of nascent transcripts. It was observed in *E. coli* during the regulation of translation of glucosamine-6-phosphate [GlcN-6-P] synthase (GlmS). In case of lack of GlmZ, the SD sequence of *glmS* mRNA is occluded in the hairpin structure. To activate the translation of *glmS* mRNA, GlmZ binds to the RBS-masking hairpin acting as an anti-antisense element and thus releasing the RBS for ribosome binding. GlmZ levels are strictly regulated by other ncRNAs, including YhbJ and GlmY, which together enable complex homeostasis of GlmS expression [40,72].

Another type of translation regulation mechanism based on RNA structure is represented by a Csr system discovered in *E. coli* (Figure 4B). The three main molecules of this system are RNA-binding protein CsrA and two sRNA, CsrB and CsrC. The homodimeric protein CsrA is a global translation inhibitor acting by binding nearby the RBS region and blocking the ribosome access to the SD sequence. CsrA binds to a specific sequence motif—GGA exposed in a loop of the hairpin structure. This process indirectly leads to the target mRNA degradation. Inseparable elements of this system regulation are already mentioned sRNAs, which both contain multiple CsrA binding sites that can sequester this protein. The transcription of *csrB* and *csrC* is regulated by a two-component signal transduction system consisting of BarA and UvrY. Interestingly CsrA can indirectly influence these sRNAs’ transcription through the response regulator UvrY. CsrA is known to inhibit glycogen synthesis, peptide transport, synthesis of a biofilm adhesin, and activate cell motility, glycolysis, and acetate metabolism. Homolog molecules working in similar systems were found also in other bacteria species, for example in *Pseudomonas fluorescens* and *Erwinia carotovora* [40,77].

## 5. Future Challenges and Perspectives

The highly dynamic nature of RNA, together with a coupling of the transcription and translation processes, provides a wide range of possible regulatory mechanisms. Although our understanding of the role of RNA secondary structure in gene expression regulation constantly grows, some aspects are still unresolved. One of the most promising recent developments which can be used for such research is related to the establishment of high-throughput structural probing techniques, such as Structure-seq, DMS-MaP, SHAPE-MaP, PARIS, and others. Similarly, as the introduction of small RNA high-throughput sequencing revealed a plethora of novel small RNAs in bacteria, a massive application of such structure probing techniques can lead to the identification of novel RNA structure-dependent regulatory mechanisms.
